# Structure and Age Jointly Influence Rates of Protein Evolution

**DOI:** 10.1371/journal.pcbi.1002542

**Published:** 2012-05-31

**Authors:** Macarena Toll-Riera, David Bostick, M. Mar Albà, Joshua B. Plotkin

**Affiliations:** 1 Evolutionary Genomics Group, Fundació Institut Municipal d'Investigació Mèdica (FIMIM)- Universitat Pompeu Fabra (UPF), Barcelona, Spain; 2 Department of Biology, University of Pennsylvania, Philadelphia, Pennsylvania, United States of America; 3 Catalan Institution for Research and Advanced Studies (ICREA), Barcelona, Spain; JCVI, United States of America

## Abstract

What factors determine a protein's rate of evolution are actively debated. Especially unclear is the relative role of intrinsic factors of present-day proteins versus historical factors such as protein age. Here we study the interplay of structural properties and evolutionary age, as determinants of protein evolutionary rate. We use a large set of one-to-one orthologs between human and mouse proteins, with mapped PDB structures. We report that previously observed structural correlations also hold within each age group – including relationships between solvent accessibility, designabililty, and evolutionary rates. However, age also plays a crucial role: age modulates the relationship between solvent accessibility and rate. Additionally, younger proteins, despite being less designable, tend to evolve faster than older proteins. We show that previously reported relationships between age and rate cannot be explained by structural biases among age groups. Finally, we introduce a knowledge-based potential function to study the stability of proteins through large-scale computation. We find that older proteins are more stable for their native structure, and more robust to mutations, than younger ones. Our results underscore that several determinants, both intrinsic and historical, can interact to determine rates of protein evolution.

## Author Summary

Rates of protein evolution vary dramatically within and between organisms. But the factors that determine a protein's evolutionary rate are still under debate, despite extensive studies over the past decade. Several determinants have been proposed, for example gene expression, the importance of the gene for the organism, the number of physical or genetic interactions it has, its structural characteristics, or when it originated. Here we study how age and structural characteristics interact with one another to influence evolutionary rates. We use a set of one-to-one orthologs of human and mouse proteins, with known crystal structures. We find that these two determinants interact: for example, the age of protein modulates how its structure correlates with evolutionary rate. Nonetheless, the influence of age on evolutionary rate cannot be explained by its interplay with structure.

## Introduction

It is well known that protein evolutionary rates are not homogeneous, with as much variation within an organism as between organisms. In fact, evolutionary rates vary as much as 1,000-fold among the proteins in the yeast *S. cerevisiae* [1]. Therefore, there has been longstanding interest in deciphering the causes of this variation, with a large literature of theoretical and empirical studies alike.

Numerous possible determinants for protein evolutionary rate have been proposed, such as protein dispensability [2], number of mRNA molecules per cell [3], [4], number of protein molecules per cell [5], codon adaptation index [4], [6], number of protein-protein interactions [7], sequence length [8], [9], role in the interaction network [10], and structural properties such as solvent accessibility and folding robustness [11]–[13]. Some of the proposed determinants are correlated with one another, which makes the identification of causal factors difficult. For this reason Drummond and colleagues [5] tried to disentangle these factors by performing a principal component regression (PCR) analysis. They found that a single component, which included codon adaptation index, protein abundance and gene expression level, accounted for nearly half of the observed variability in protein's evolution. Nonetheless, those expression-related factors have been measured with less noise than other possible factors. This further complicates even the principal component regression [14]. In related work, Drummond and Wilke [15] observed covariation between sequence evolution, codon usage and mRNA level among a broad range of species. They suggested there may be selection for robustness against mistranslation, since mistranslation-induced misfolding would be more deleterious for highly expressed proteins.

A protein's three-dimensional structure may also be a key factor in determining its evolutionary rate. The core of a protein is mostly formed by buried residues, which often play a crucial role in the stability of the folded structure [16]. Most mutations in the core of a protein tend to destabilize the protein [17]. Exposed residues are in contact with solvent and they are known to evolve faster than buried ones [11], [12], [18]–[21]. In fact, the more general relationship between solvent exposure and evolutionary rate is linear and very strong [12]. Given these results, we might expect those proteins with a higher fraction of exposed residues to evolve faster. But, surprisingly, Bloom and others found the contrary pattern [11], [12]. Bloom et al explained this incongruence using protein designability, defined roughly as the number of sequences than can fold into a structure. Since a higher number of sequences can fold into highly designable structures, designable structures are more tolerant to mutations and hence, evolve faster. As designability has been related to contact density [22] and contact density is highly correlated with the fraction of buried residues, the authors hypothesize that highly designable proteins have a higher fraction of buried residues. Consequently, highly designable proteins have stable core, allowing the exposed residues to freely mutate without compromising stability. In fact, Franzosa and Xia [12] have demonstrated how large-core proteins (which are the ones having an overall low solvent exposure value) have low solvent exposure values but high *d*_N_/*d*_S_, specially observing that highly exposed residues in large-core proteins are evolving faster than in small-core proteins. Also, proteins with a higher contact density tend to evolve more rapidly – in fly, yeast, *E.coli* and human [23]. Moreover, highly designable proteins have been shown to evolve more functional innovations [24]. Bloom and colleagues [11] have carried out a PCR analysis showing that the component measuring expression level could explain around 34% of the rate variation, whereas structural characteristics explained approximately the 10% of the rate variation. There are other structural properties correlated with evolutionary rates, such as the number of intra-protein residue interactions, which tend to reduce rates of evolution [25]. Structure itself could be a determinant of protein evolution, or indeed, could be acting through other mechanisms. For example, it could play a crucial role in the selection for structural robustness against mistranslation in highly expressed proteins, which has already been shown to be a key determinant of protein evolution [11].

Quite aside from the factors discussed above, which are intrinsic to the properties of a protein in an organism today, studies have also shown that the age of a protein, which depends on its evolutionary history, is also correlated with evolutionary rates [26]–[28]. In particular, an inverse relationship between age and evolutionary rate has been widely observed [26], [27], [29], suggesting that a protein's evolution could be shaped in part by its evolutionary origin. This relationship has been reported in a broad range of organisms: primates [30], mammals [26], *Drosophila* [27], [29], *Plasmodium* [31], fungi [32] and bacteria [33].

Despite all these findings, what factors determine a protein's evolutionary rate are still under debate – and the relative role of intrinsic factors of present-day proteins, versus historical factors such as protein age, remains poorly characterized. Here we explored the interplay between two very different factors: a protein's age and its structural properties. Our objective is to determine whether structural biases among age groups could explain the reported differences in evolutionary rates with age [26], [27]. To do so we used a dataset of human proteins with homologues in mouse for which we were able to map a PDB structure. Age was assigned to each PDB structure and then structural properties (solvent exposure, designability, stability and secondary structure) were calculated among the PDB structures classified in the age groups. We found that differences in evolutionary rates previously observed among age groups could not be explained due to differences in the structural properties among age groups. Similarly, differences in rates correlated with structural differences cannot be entirely explained by the age of the PDB structure, although a marginal influence of age is observed. Our results therefore reinforce the idea that there is not a single determinant of evolutionary rate, and that both intrinsic present-day properties as well as evolutionary age independently contribute to differential rates of protein evolution.

## Results

### Interactions between age and structural determinants of evolutionary rates

It has been widely argued that both protein structure and protein age play important roles as determinants of protein evolution. However, how structure and protein age are related has not been yet studied. We have found an interesting interplay between structure and age: a set of structural characteristics that are correlated with evolutionary rates but in a manner that depends on protein age.

#### Linear relationship between relative solvent accessibility and evolutionary rate

We calculated the relative solvent accessibility (RSA) for each residue in every PDB structure that mapped to human proteins (406,970 residues in total, across 2,595 PDB structures). We apportioned the RSA values into 20 bins and we concatenated all the residues within each bin to calculate the evolutionary rate (measured as *d*_N_) of residues as a function of accessibility. We found a strong correlation between RSA and *d*_N_ (Pearson correlation: 0.971, p-value = 1.179 e^−12^) in mammals (Figure S1). A similar linear correlation between evolutionary rate and RSA was previously reported in yeast [12], suggesting that this relationship is an universal trend.

**Figure 1:**
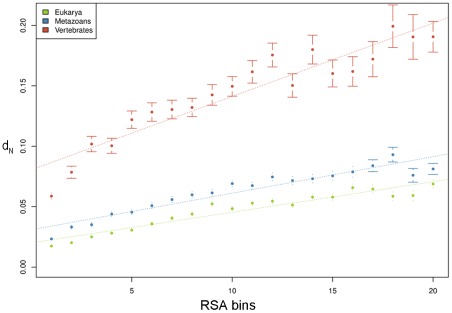
Linear relationship between solvent accessibility and *d*_N_ in Eukarya, Metazoans and Vertebrates age groups. Eukarya: Pearson correlation: 0.957, p-value = 4.477e^−11^; Metazoans: Pearson correlation: 0.950, p-value = 1.445e^−10^; Vertebrates: Pearson correlation: 0.941, p-value = 7.005e^−10^. Errors bars indicate the standard error for the *d*_N_ calculation.

Additionally, we separated the PDB structures according to their age (i.e. the youngest proteins, which originated in Vertebrates, the medium-aged proteins which originated in Metazoans, and the oldest proteins which originated in Eukaryotes) and we found a similar correlation between RSA and evolutionary rate within each age group (Pearson correlation >0.94 and p-value<10^−10^ in all the age groups) (Figure 1). But, interestingly, the slope is different among age groups: the younger proteins show a more dramatic influence of RSA on evolutionary rate. For the linear model *d*_N_∼RSA, the slope in Eukarya is 0.0025; for Metazoans and Vertebrates, it is 0.003 and 0.006, respectively. We also considered an interaction term of RSA with age (*d*_N_∼RSA+RSA*age+age) in all the possible pairwise comparisons between age groups, in order to assess the importance of age. The interaction was generally significant (Eukarya vs Metazoans: 0.11, Eukarya vs Vertebrates: 1.70e-^07^, Metazoans vs Vertebrates: 4.73e^−06^) supporting the notion that age plays a role in shaping the relationship between solvent accessibility and evolutionary rate.

#### Fraction of residues exposed and designability

Given the linear relationship between solvent accessibility and evolutionary rates one expects to find that those structures containing a higher number of exposed residues would be evolving faster. But Bloom and colleagues [11] have found exactly the contrary: the fraction of buried residues in a protein is positively correlated with its evolutionary rate (*d*_N_). Bloom et al explained this incongruence using the concept of protein designability, as discussed above. Here we have been more stringent than in earlier studies, using 99% sequence identity to assign structure as compared with the 40% criteria used in Zhou et al [23].

**Figure 2:**
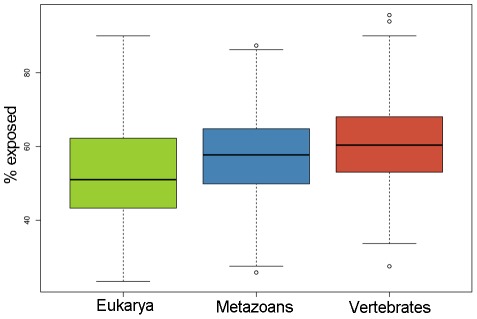
Percentage of residues exposed in PDB structures classified in 3 age groups: Eukarya, Metazoans and Vertebrates. Wilcoxon tests were performed to assess the significance of the difference: Eukarya vs Metazoans: p-value<2.2e^−16^ , Eukarya vs Vertebrate: p-value<2.2e^−16^ , Metazoans vs Vertebrates: p-value = 0.0005 ).

We tested the impact of designability in the context of PDB structures classified by their age of origin. We first calculated the evolutionary rate (*d*_N_) of each PDB structure as well as the fraction of residues exposed (exposed residues/(buried+exposed residues) *100). We found that the oldest Eukaryotic PDB structures were evolving the slowest, followed by Metazoans and then Vertebrates (Wilcoxon tests, p-value<10^−3^ in all the pairwise comparisons). This confirms the inverse relationship between protein age and evolutionary rate that has been reported previously [26] (Figure S2). Besides, older folds have been previously reported to be more conserved than younger ones [34]. At the same time, we found that younger PDB structures have a significantly higher fraction of exposed residues than older ones (Wilcoxon tests, p-value<10^−3^ in all the pairwise comparisons) (Figure 2), despite the fact that the younger PDB structures evolve faster. This is contradictory with what has been found in Bloom et al. [11] and Franzosa et al. [12].

In an effort to disentangle this contradictory result we obtained for each age group the fastest (*d*_N_/*d*_S_>0.1) and the slowest evolving PDB structures (*d*_N_/*d*_S_ = 0.001 in Eukarya and Metazoan and *d*_N_/*d*_S_<0.1 in Vertebrates) and we checked their fraction of exposed residues. Within the three age groups we found that the fastest evolving PDB structures had a higher fraction of buried residues than the slowest ones (Wilcoxon test, Eukarya: p-value = 2.697e^−07^, Metazoans: p-value = 0.004, Vertebrates: p-value = 0.05). Furthermore, among the fastest evolving PDB structures, the younger ones had a lower fraction of buried residues than the older ones (Wilcoxon test, Eukarya vs Metazoans: p-value = 2.765e^−05^, Eukarya vs Vertebrates: p-value = 2.140e^−10^, Metazoans vs Vertebrats: p-value = 0.0008). Thus, while the impact of designability on evolutionary rate holds within each age class, it does not hold between age groups. Therefore, our results in part confirm those of Bloom et al. [11], at least within each age class, but they also suggest that protein age has a stronger overall relationship with evolutionary rate than designability does.

### Protein age, stability, and mutational robustness

An important, related question is whether protein stability depends on protein age. To quantify stability for the large set of proteins used in this study, we used a well-known coarse-grained four-body knowledge-based potential function (see Materials and Methods), described by Gan [35] and Krishnamoorthy [36]. This potential has been shown to successfully score stability changes due to both mutational and structural protein alterations in a manner consistent with free energy changes derived from unfolding experiments [37], [38]. Thus, for convenience in what follows, we refer to the score of a given protein (conformation+sequence) as ΔG (analogous to the free energy of folding: lower ΔG implies greater stability). To validate our implementation of this potential, we tested its ability to distinguish native from misfolded decoy protein conformations (i.e., physically reasonable alternative protein conformations generated computationally from a native structure) taken from a standard database [39].Our implementation of the score ranked native structures among their decoys in a manner consistent with (in some cases, more favorably than) previous work [36] (Table S1).

As a secondary validation of our stability scoring function, we re-considered the correlation between RSA and evolutionary rate, described above. Given this empirical correlation, we should expect that mutations with a higher impact on the stability of the protein would tend to occur in the residues that are more buried. To test this computationally, for every protein in our PDB data set, we mutated each residue to a randomly selected residue while holding all other residue identities fixed. Then, we classified each residue in a bin according to the impact of the mutation on the stability score relative to the native sequence (using the absolute value, |ΔΔG|, where |ΔΔG| = |ΔG (native)−ΔG (mutant)|−larger values imply greater absolute perturbations to the stability). We found that the residues with less solvent accessibility exhibited significantly greater impacts on computed stability when mutated, in accordance with expectation (Figure S3).

We used the potential function to score the overall stability, measured as ΔG, for each PDB structure. To control for any length dependence in the score (a correlation between length and contact density has already been reported [11]), we binned the lengths of all structures to obtain a set of structures with the exact same length distribution within each age class. In doing so, however, we were not able to retain enough Vertebrate PDB structures for further analysis, and so restricted our comparisons to Eukarya and Metazoans. When we compared ΔG amongst Eukarya and Metazoans, paired by length bin, we found that Eukaryotic structures are more stable on average (Wilcoxon-paired test, p-value<0.01, Eukarya median: −90.74, Metazoan median: −85.08). This suggests that older proteins are more stable, on average, than younger proteins.

**Figure 3:**
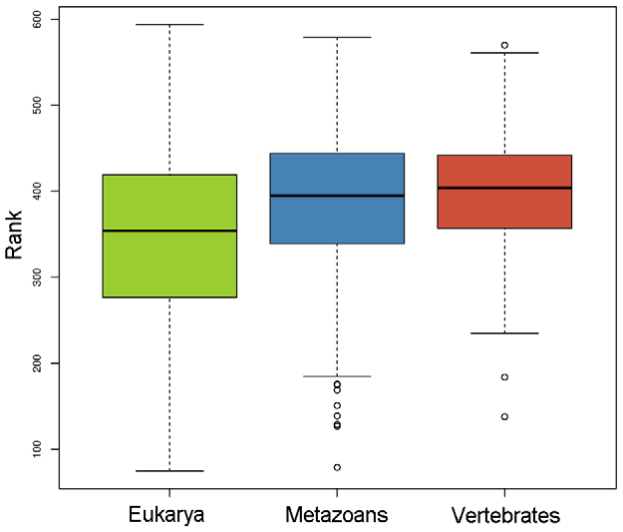
Rank of the stability score of wildtype protein sequence among 1000 mutated sequences in 3 age groups: Eukarya, Metazoans and Vertebrates. Wilcoxon tests were performed to assess the significance of the difference: Eukarya vs Metazoans: p-value<1.684e^−14^ , Eukarya vs Vertebrate: p-value<2.2e^−16^ , Metazoans vs Vertebrates: p-value = 1.119e^−8^). Low rank suggests that the native structure is relatively robust to mutations.

Furthermore, we studied how mutational robustness might vary with protein age. To estimate robustness we simulated random amino-acid mutations in 2% of the residues of each PDB structure, and we repeated this process 1000 times for each structure (Figure S4). We then used two measures, Z-score and Rank, to assess how robust the native structure is to mutation. The Z-score was calculated for each protein as the protein's stability score minus the mean score for the population of 1000 mutated structures divided by its standard deviation, σ, (Z = (ΔG−〈ΔG〉/σ). Younger PDB structures were significantly less robust to mutations (higher Z-score) than older proteins (Wilcoxon test, Eukarya vs Metazoans p-value<10^−15^, Eukarya vs Vertebrates p-value<10^−14^, Metazoans vs Vertebrates p-value = 0.131). We also computed the rank of each native protein score within the population of 1000 mutant scores. We found the same trend: the native sequence-structure compatibility of younger proteins was significantly less robust (higher rank) than that of older proteins (Wilcoxon test, p-value<10^−7^ in all the pairwise comparisons) (Figure 3). Similar results were obtained when we increased the mutation rate to 10% of residues within each PDB structure (data not shown).

More designable proteins are generally more stable [40] and have a higher fraction of buried residues, which may lead to a more robust protein core. It has been shown that stability generally enhances tolerance to mutations – more beneficial mutations are accepted because they do not destabilize the native structure [41], [42]. Thus, our results on the greater stability and robustness of older proteins generally concord with earlier notions of designability and mutational tolerance.

### Protein age and secondary structure

We also investigated the relationships between protein age, secondary structure classification, and evolutionary rates. We classified each residue in every PDB structure according to the type of secondary structure in which it participates as well as according to whether it is buried (RSA<25%) or exposed (RSA>25%) as in Bloom et al. [11]. Each residue was mapped to one of four secondary structure categories by DSSP [43]: helix (class H in DSSP), sheet (class E in DSSP), turn (classes S and T), coil (classes B, G, I and “.”). Evolutionary rates within each structural category were computed by concatenating, for each PDB structure, all the residues classified in a given structural category and comparing those residue positions to homologous positions in mouse.

**Figure 4:**
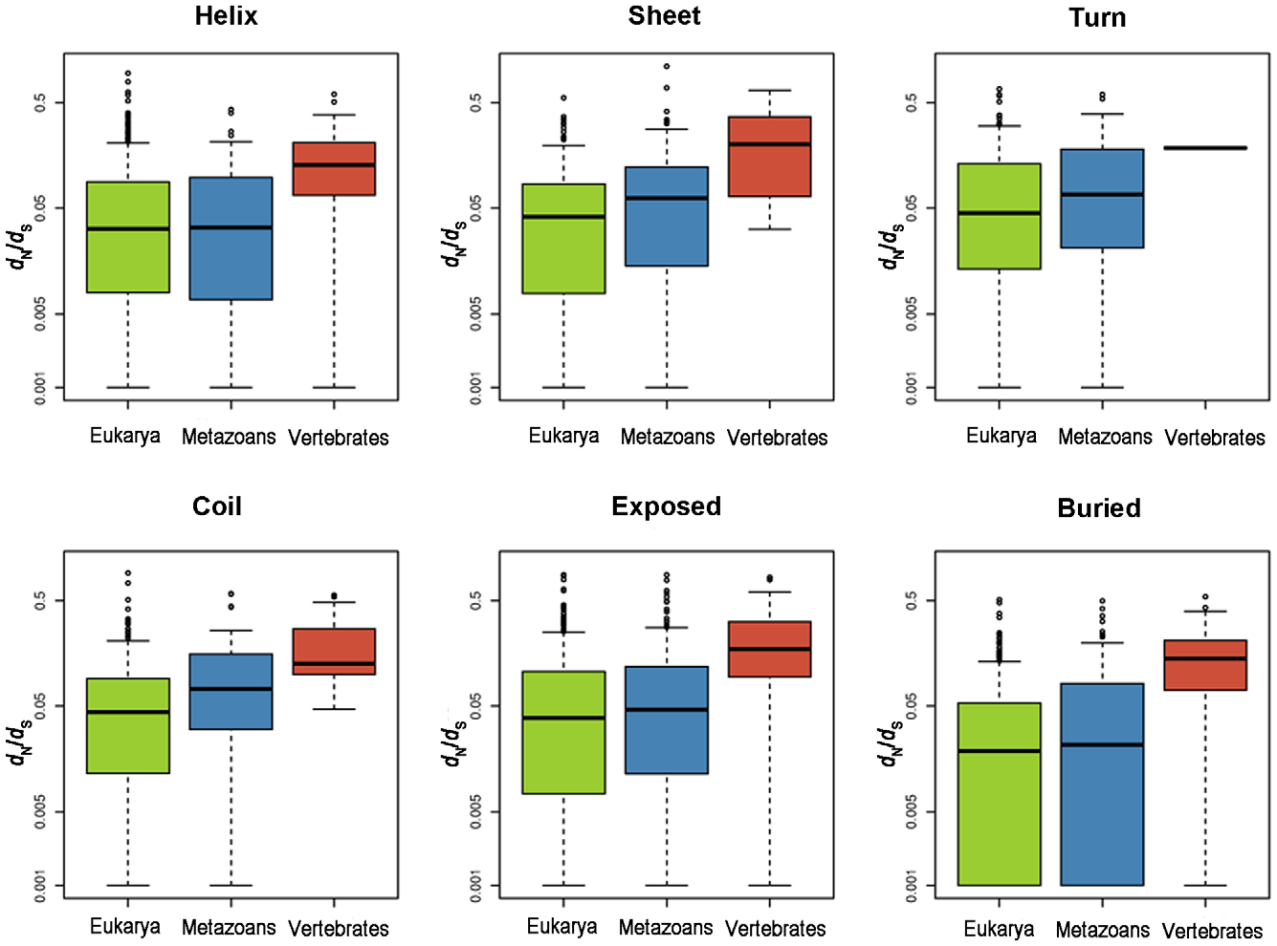
Evolutionary rates by age and secondary structure/solvent accessibility categories. An inverse correlation between the age of the protein and evolutionary rate occurs within each structural category. Wilcoxon tests were performed (see Table 1).

Generally, we found that exposed residues evolved faster than buried ones (Wilcoxon test, p-value<0.01) and that residues classified as helix evolve slower (Wilcoxon test, p-value<0.01) than the residues classified in other categories (Figure S5). More importantly, when we separated the secondary structures and solvent accessibility according to age group we found that the younger structures were evolving faster than the older ones (Wilcoxon test, Table 1, Figure 4) within each structural category. This implies that differences in the frequency of structural categories by age class cannot explain the previously reported inverse relationship between protein age and evolutionary rate [26]. Thus, this analysis supports the important role for protein age in shaping evolutionary rates, above and beyond the influence of solvent accessibility and secondary structure.

**Table 1:** Comparisons between the 3 age classes in each secondary structure and solvent accessibility types.

Secondary structure	Age	*d*_N_/*d*_S_	*d*_N_
Helix	Eukarya-Metazoan	0.929	0.600
	Eukarya-Vertebrates	5.286e^−06^	5.188e^−06^
	Metazoans-Vertebrates	4.771e^−05^	5.74e^−05^
Sheet	Eukarya-Metazoan	0.048	0.009
	Eukarya-Vertebrates	2.737e^−08^	2.521e^−09^
	Metazoans-Vertebrates	3.057e^−05^	4.129e^−05^
Turn	Eukarya-Metazoan	0.4841	0.205
Coil	Eukarya-Metazoan	0.001	0.0002
	Eukarya-Vertebrates	3.070e^−05^	3.542e^−06^
	Metazoans-Vertebrates	0.010	0.005
Exposed	Eukarya-Metazoan	0.132	0.010
	Eukarya-Vertebrates	2.681e^−16^	<2.2e^−16^
	Metazoans-Vertebrates	7.402e^−13^	4.318e^−12^
Buried	Eukarya-Metazoan	0.066	0.005
	Eukarya-Vertebrates	<2.2e^−16^	<2.2e^−16^
	Metazoans-Vertebrates	3.713e^−12^	4.207e^−12^

## Discussion

Interactions among various determinants of protein evolution are not well understood despite several decades of investigation. In this work, we have studied two types of proposed determinants: structural properties intrinsic to present-day proteins, and protein age. We found that several well-known relationships between structural properties and evolutionary rate that had previously been reported, regardless of age, also hold within each age class: residues with high solvent accessibility evolve more quickly [11], [12], [18]–[21], while proteins with a larger fraction of exposed residues evolve more slowly [11], [12]. At the same time, the age of a protein can modulate the correlation between structural properties and evolutionary rates – e.g. the strength of relationship between solvent accessibility and evolutionary rate depends on the age of the protein in which the residue is found. We also studied secondary structures of proteins, and we confirmed that the typical inverse relationship between protein age and evolutionary rate holds within each structural class of residues. This implies that differences in the frequency of structural categories by age class cannot explain the previously reported inverse relationship between age and rate. Finally, we introduced a knowledge-based potential to study the relationships between protein age and stability. We found that older proteins are more stable, on average, than younger proteins, and that older structures are also more robust to mutation than younger structures.

Our results provide a more nuanced view on the determinants of protein evolutionary rates. Whereas some determinants of rates hold within each age class, age can nonetheless modulate these effects. And other relationships that hold regardless of age (such as, proteins with a greater fraction of exposed residues evolve more slowly) cannot explain differences in rates among age classes.

Our analyses certainly suffer from several drawbacks. Most important, we were able to map a structure to only 14% of the one-to-one orthologs proteins between human and mouse. This fraction would be even smaller if we had chosen other species. Despite the increase in solved structures over the past few years, the number of mapped structures is still a small fraction of known proteins. Additionally, we have to bear in mind that there are biases in the type of proteins that enjoy solved structures. For example disordered regions are poorly represented in PDB, as they are difficult to crystallize. Younger proteins are enriched in low-complexity regions [44], [45], many of which are expected to be disordered [45]. How this adds to the differences in evolutionary rates among age classes is an aspect that remains to be studied.

Choi and Kim [46] have reported that old proteins are longer and have more complex tertiary structures (α/β) than younger proteins, hypothesizing that proteins tend to become more complex in their structure along their evolutionary history. Our results also give insights on the evolution of protein structural characteristics, as we have found that older structures are more designable, stable and robust to mutations than younger ones. These findings suggest that structures may acquire stability and robustness to mutations with time. However, these findings also raise new questions. Since stability increases a protein's tolerance to mutations [42] we might expect that younger structures would be evolving slowly due to the destabilizing effect of mutations. But we find them to evolve fast. One possible explanation is that previous studies have assumed proteins are generally under the same degree of selection, regardless of age. But some of our results might be due to differential strengths of selection in old versus young proteins. One possibility is that younger sequences mapped to PDB may be experiencing strong positive selection for stabilizing mutations, which explains their higher rates of evolution; whereas older protein are already stable and robust, and thus lack this type of positive selection. However, using single nucleotide polymorphism (SNP) data Cai and Petrov have found limited evidence for increased positive selection in primate-specific genes, and strong indications that relaxed negative selection is likely to be more important in young genes than in older genes [47]. Therefore, it may be that selection for high stability is reduced in younger proteins. In conclusion, our results reinforce the idea that protein evolution is not explained by a single determinant, but rather by the interplay of many determinants, including even factors that are not intrinsic to the present-day protein but depend on evolutionary age.

## Materials and Methods

### Datasets

13494 orthologs one-to-one between *Homo sapiens* and *Mus musculus* were obtained from Ensembl (version 62) [48]. In order to assign a known structure to our proteins we performed BlastP searches [49] between the structures deposited in the Protein Data Bank [50] and our dataset of human proteins with orthologs in mouse. We only kept those hits with an identity at least of 99%. If several hits were overlapping we chose the one that is closer to the human protein. Afterwards we applied a strong filtering process in which we discarded 506 PDB structures because they were either shorter than 50 amino acids, had a discontinuous (gapped) chain, or had an incomplete backbone structure. After discarding these structures we were left with 1,899 proteins with at least one PDB structure mapped to them, encompassing a total of 2,145 structures.

For each human protein region with a structure assigned we recorded the information regarding to the solvent-accessibility and the secondary structure. The information for the secondary structure and for solvent accessibility was obtained from the DSSP files (downloaded from http://srs.ebi.ac.uk/srsbin/cgi-bin/wgetz?-pageLibInfo-libDSSP). We only recorded those positions in which there was the same amino acid in the human protein and in the PDB structure. Residues were classified in 4 secondary structures based on the DSSP [43] assignation for the residue: helix (class H in DSSP), sheet (class E in DSSP), turn (classes S and T) and coil (classes B, G, I and “.”), as in Bloom et al. [11]. For each residue we calculated the solvent-accessibility as the RSA (relative solvent accessibility). RSA was obtained normalizing the accessibility obtained from DSSP by the reference solvent-accessible surface areas (ASA) of each amino acid. ASA is calculated for residue X in an extended Gly-X-Gly peptide; ASA values were obtained from Miller et al. [51]. Some residues were found to have RSA>1. We treated those cases as if they had RSA = 1, as several earlier studies have done [12], [52]. Residues were classified as buried if the RSA value was lower than 25% and as exposed if it was higher than 25%, as in Bloom et al. [11]. Additionally we binned the RSA values in 20 bins, and we classified each residue in one of these RSA bins.

The fraction of exposed residues for a given PDB was calculated dividing the number of residues classified as exposed by the sum of the number of exposed and buried residues.

### Age assignation

For each PDB structure we used BlastP searches with an e-value cut-off of 10^−4^ against several genomes to asses the presence of homologues. We used the following age classes: mammals (*Mus musculus*, *Rattus norvegicus*), non-mamalian vertebrates (*Gallus gallus*, *Xenopus tropicalis*, *Danio rerio*, *Takifugu rubripes*), other metazoans (*Ciona intestinalis*, *Drosophila melanogaser*, *Anopheles gambiae*, *Caenorhabditis elegans*) and other eukaryotes (*Schizosaccharomyces pombe*, *Saccharomyces cerevisiae*, *Oryza sativa*, *Arabidopsis thaliana*). Then, an age is assigned to each PDB chain according to the phylogenetic width of its homologues. We obtained 1157 PDB structures classified as eukarya, 725 as metazoan, 253 as vertebrate and 25 as mammals. As very few PDB structures were classified as mammals they were discarded for the analysis.

### Evolutionary rates estimation

To estimate the evolutionary rates we only used those PDB structures in which the corresponding region in the human protein had at least 50% identity with its syntenic region in mouse. Pairwise alignments for the protein region corresponding to the PDB structure in human and in mouse were performed using T-Coffee [53] and subsequently we obtained the nucleotide coding sequence alignment using an in-house Perl program.

To perform the secondary structure and the solvent-accessibility analysis we concatenated for each PDB region in the protein all the residues that were sharing the same type of secondary structure/solvent-accessibility, as long as the amino acid position in the protein was exactly the same as in the PDB structure. Then, for example, for a given protein region with a mapped PDB structure, we concatenated all the residues that were classified as helix and we took also the corresponding residues in mouse (as long as the mouse region homologous to human and human had at least a 50% of identity, which was accomplished in the majority of the cases). Therefore, we constructed two new orthologous sequences with information corresponding only to one type of structure, helix in this case. These new sequences were aligned using T-coffee and realigned afterwards at nucleotide coding sequence level.

We additionally concatenated all the PDB residues classified in the same RSA bin and also all the residues that were classified in the same RSA bin and in the same age. The corresponding residue in mouse was also obtained. By doing that we obtained very long orthologous sequences that were aligned using MAFFT [54].

To estimate the evolutionary rates we calculated the number of non-synonymous substitutions per non-synonymous site (*d*_N_), the number of synonymous substitutions per synonymous site (*d*_S_) and the *d*_N_/*d*_S_ ratio using the codeml program, which is inside the PAML software packages [55].

Several filters have been applied to the evolutionary rates estimations to ensure their robustness. Sequences shorter than 60 amino acids were discarded, as well as sequences with *d*_N_>0.5 and/or *d*_S_>2 which could be indicative of a lack of homology and of the presence of sequence saturation respectively.

### Stability computations

To calculate the stability of the PDB structures we used a knowledge based potential, described by Gan [35] and Krishnamoorthy [36]. The potential function was trained on a non-redundant set of 3,425 X-ray protein structures downloaded from the PISCES database [56] maintained by the Dunbrack laboratory. This set of proteins represented a subset of a list of 4,944 PDB chains that met strict parsing criteria [36]. Each chain in the set shares no more than 25% sequence identity with any other chain, was resolved to <2.0 Angstroms, and solved with an R-factor of 0.25 or better. This type of potential has been widely validated [37], [57].

We did two rounds of point mutations. In the first round we introduced 1 random mutation with random placement along the sequence for every 50 amino acids in the protein. In the second round, 1 random mutation with random placement along the sequence for every 10 amino acids. We repeated this process 1000 times for each PDB structure, obtaining 1000 mutated structures. For those structures obtained by NMR spectroscopy we used the first structural model presented in the PDB file. Then, we assessed the stability for the native PDB structure and mutated sequence using the potential, obtaining the measure, ΔG, which describes the stability – lesser values imply more stability. We also calculated the destabilizing effect of mutations (robustness) using Z-score and Rank measures. The Z-score for a protein structure with specified sequence is calculated as (Z = (ΔG−〈ΔG〉/σ), where 〈ΔG〉 is the average stability score and σ is the standard deviation in ΔG derived from the 1000 mutated structures. The rank of the native sequence in these experiments is defined as the enumerated position of the native ΔG value in the sorted list – from lowest (most stable) to highest (least stable) – of ΔG values from the 1000 mutated structures.

To control for any possible dependence of the knowledge based potential score on protein length, we binned the PDB structures in our data set by length when comparing native ΔG values for the proteins classified by age. In doing so, we ensure that our comparisons of stability across age grouped proteins are unbiased by protein length. Due to this binning, we lacked sufficient data to perform these comparisons for the representative Vertebrate PDB structures.

## Supporting Information

Figure S1 **Linear correlation between** ***d*** **_N_ and solvent accessibility (RSA).** Pearson correlation: 0.971, p-value = 1.179 e^−12^. RSA was separated in 20 bins and residues classified in the same bin were concatenated for all the PDBs to calculate the evolutionary rates.
(TIF)

Figure S2 **Evolutionary rates (measured as** ***d*** **_N_/** ***d*** **_S_) in the three age groups: Eukarya, Metazoans, Vertebrates.** The differences are significant in all pairwise comparisons (wilcoxon tests, Eukarya vs Metazoans: p-value = 0.004, Eukarya vs Vertebrates: p-value<2.2e^−16^ , Metazoans vs Vertebrates: p-value<2.2e^−16^ ).
(TIF)

Figure S3 **Mutations with a higher impact tend to occur in more buried residues.** Differences between delta delta G are highly significant (wilcoxon test, p-value<2.2 e^−16^) except for the comparison between bin 6 and 7 and bin 7 and 8.
(TIF)

Figure S4 **Diagram representing the pipeline done to assess PDB's robustness against point mutations.**
(TIF)

Figure S5 **Residues classified in structural classes (Helix, Sheet, Turn and Coil) and solvent accessibility properties (Buried, Exposed).** Two trends could be observed 1) exposed residues evolve faster than buried ones (wilcoxon test, p-value<0.01), 2) helix structure is evolving slower than the other types of secondary structures (wilcoxon test, p-value<0.01).
(TIF)

Table S1 **Structure recognition: Discrimination of native from decoy structures.** Comparison of the performance of our potential (Native rank) with the performance of the potential derived by Feng [58] and Krishnamoorthy [36].
(PDF)
