# Supplementary figures and images for: Structure and Age Jointly Influence Rates of Protein Evolution
Source: PLoS Comput Biol. 2012 May 31;8(5):e1002542. doi: 10.1371/journal.pcbi.1002542 (PMC3364943; doi:10.1371/journal.pcbi.1002542)

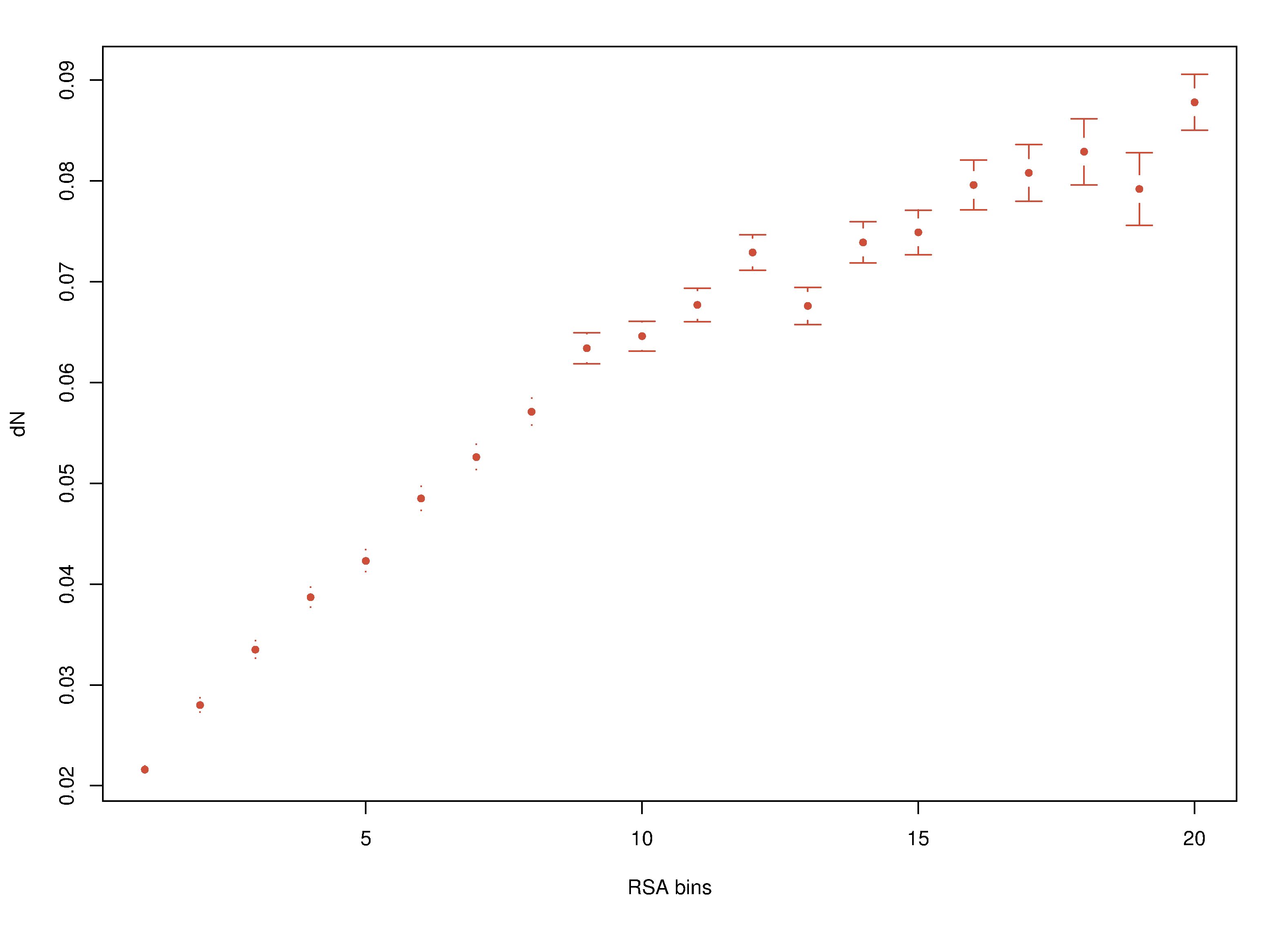

Supplement: Figure S1 — Linear correlation between d N and solvent accessibility (RSA). Pearson correlation: 0.971, p-value = 1.179 e−12. RSA was separated in 20 bins and residues classified in the same bin were concatenated for all the PDBs to calculate the evolutionary rates. (TIF) [file pcbi.1002542.s001.tif]

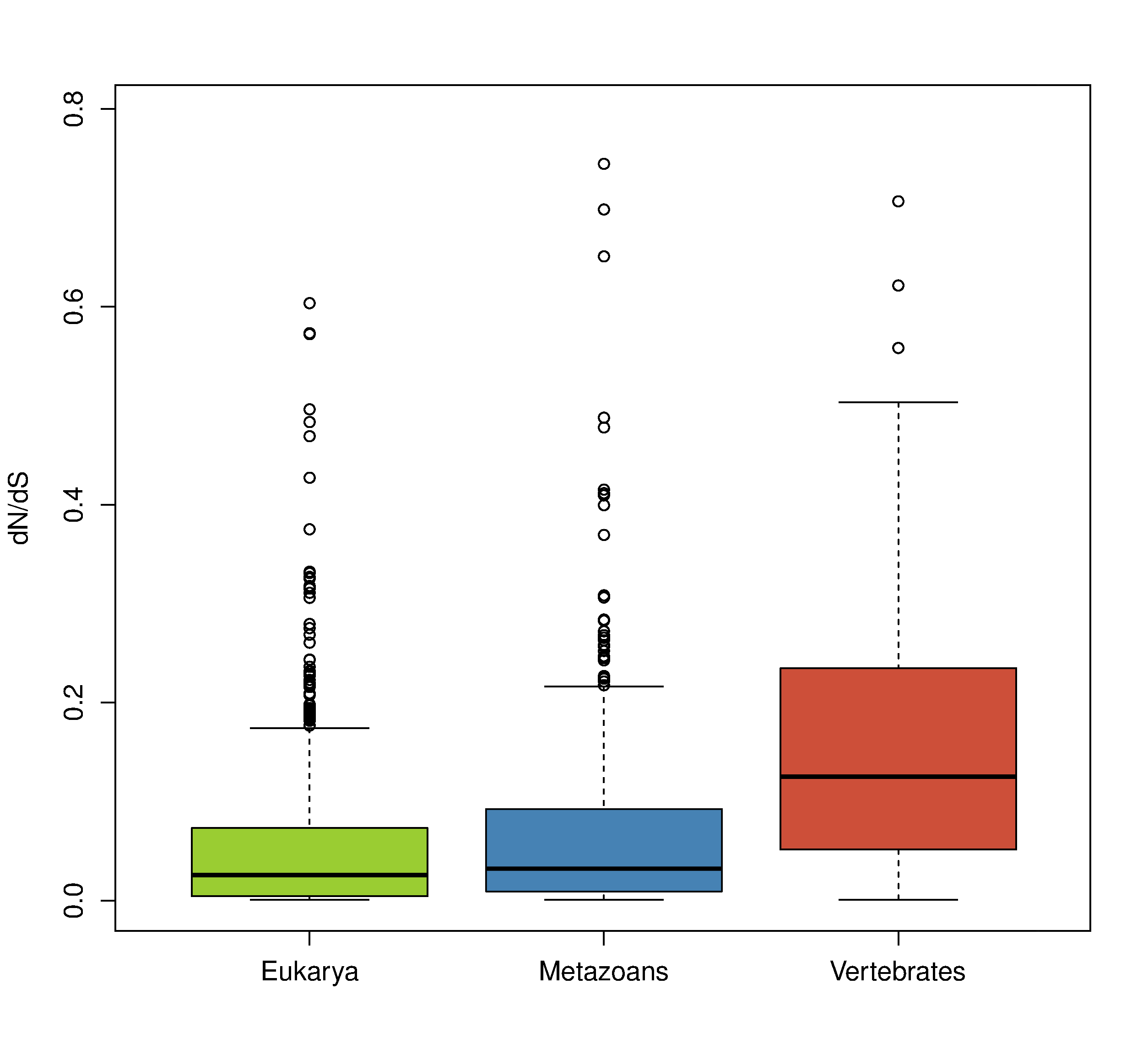

Supplement: Figure S2 — Evolutionary rates (measured as d N/ d S) in the three age groups: Eukarya, Metazoans, Vertebrates. The differences are significant in all pairwise comparisons (wilcoxon tests, Eukarya vs Metazoans: p-value = 0.004, Eukarya vs Vertebrates: p-value<2.2e−16 , Metazoans vs Vertebrates: p-value<2.2e−16 ). (TIF) [file pcbi.1002542.s002.tif]

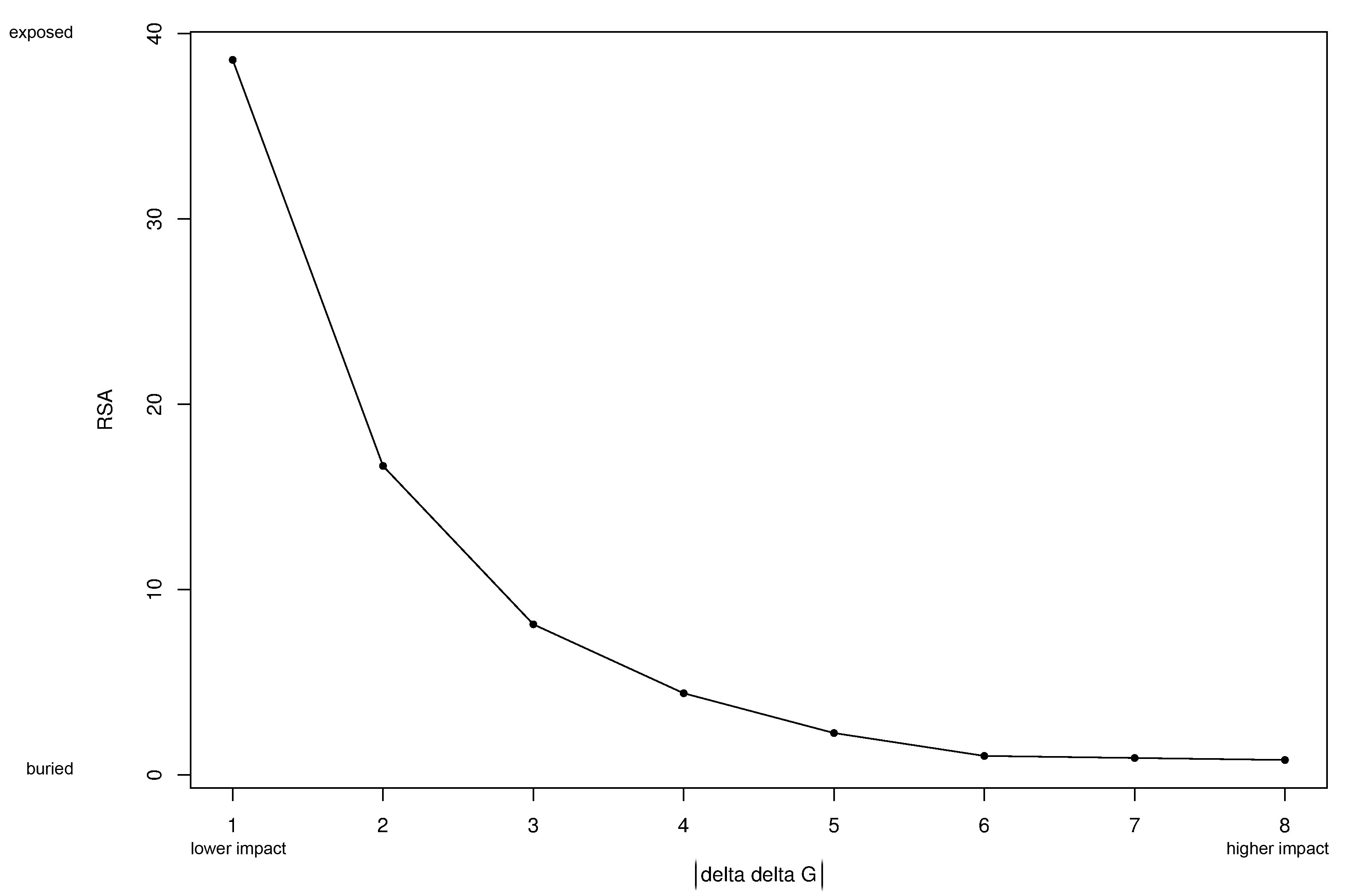

Supplement: Figure S3 — Mutations with a higher impact tend to occur in more buried residues. Differences between delta delta G are highly significant (wilcoxon test, p-value<2.2 e−16) except for the comparison between bin 6 and 7 and bin 7 and 8. (TIF) [file pcbi.1002542.s003.tif]

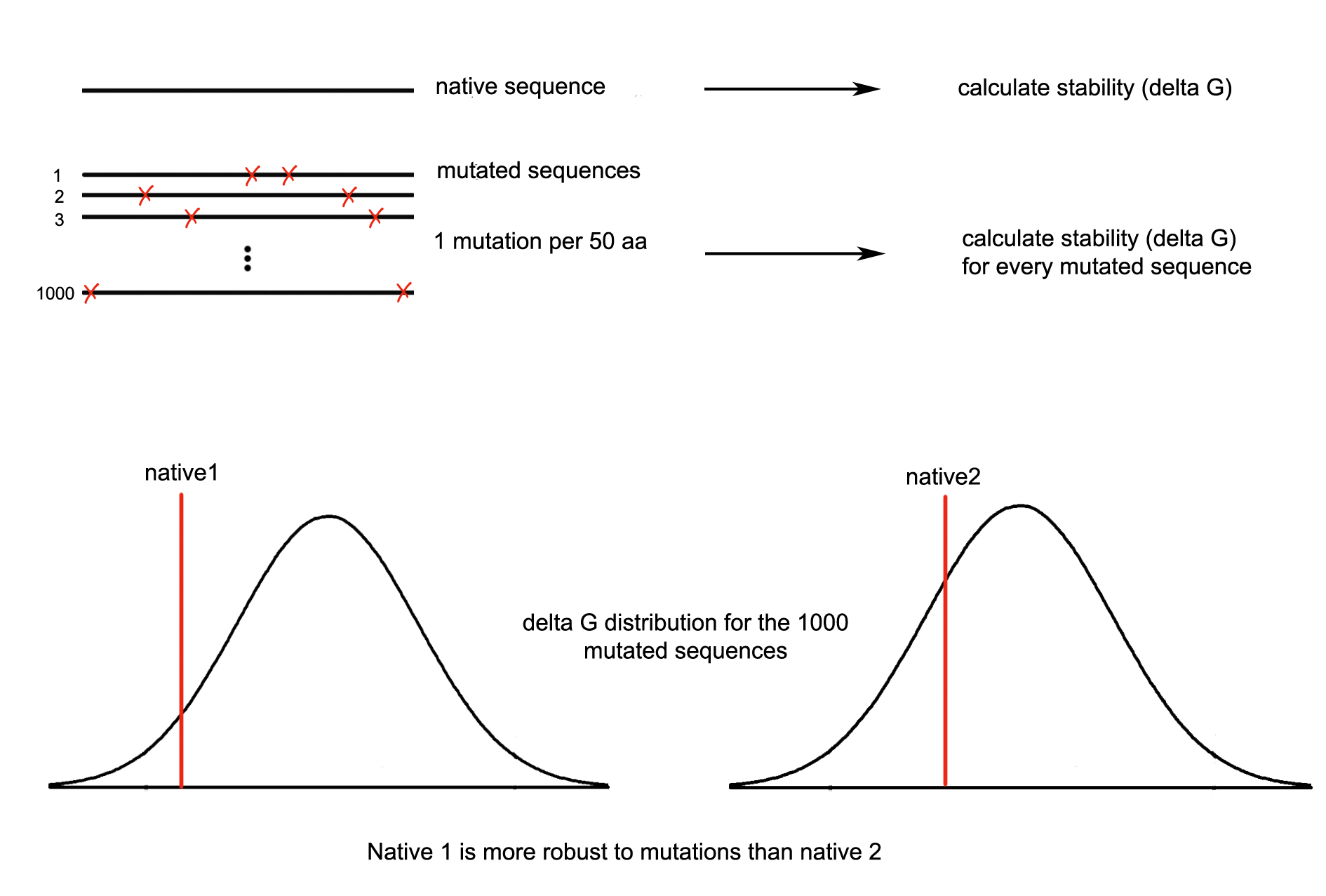

Supplement: Figure S4 — Diagram representing the pipeline done to assess PDB's robustness against point mutations. (TIF) [file pcbi.1002542.s004.tif]

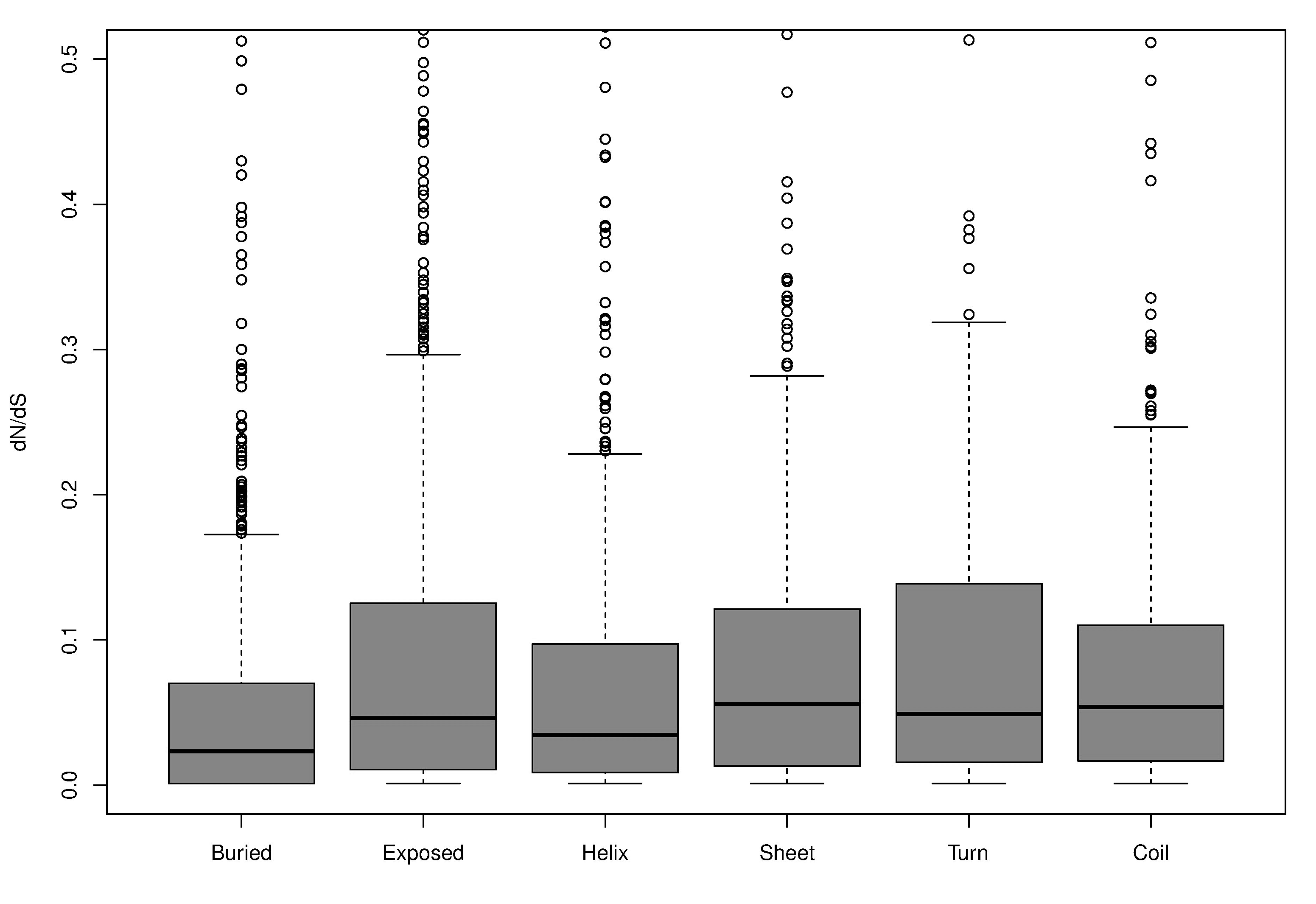

Supplement: Figure S5 — Residues classified in structural classes (Helix, Sheet, Turn and Coil) and solvent accessibility properties (Buried, Exposed). Two trends could be observed 1) exposed residues evolve faster than buried ones (wilcoxon test, p-value<0.01), 2) helix structure is evolving slower than the other types of secondary structures (wilcoxon test, p-value<0.01). (TIF) [file pcbi.1002542.s005.tif]
