# Supplementary material for: Structure and Age Jointly Influence Rates of Protein Evolution
Source: PLoS Comput Biol. 2012 May 31;8(5):e1002542. doi: 10.1371/journal.pcbi.1002542 (PMC3364943; doi:10.1371/journal.pcbi.1002542)
Supplement: Table S1 — Structure recognition: Discrimination of native from decoy structures. Comparison of the performance of our potential (Native rank) with the performance of the potential derived by Feng [58] and Krishnamoorthy [36]. (PDF) [file pcbi.1002542.s006.pdf]

Protein	Native Rank	Native Z-score	Native Rank (Feng)	Native Rank (Krishnamoorthy)
1beo	1 (1998)	-6.6	1	1
1ctf	1 (654)	-5.5	1	1
1dkt-A	1 (693)	-4.5	19	89
1fca	1 (2001)	-6.6	301	1
1nkl	1 (1995)	-7.7	1	1
1pgb	1 (1995)	-5.7	39	14
1trl-A	142 (1995)	-1.5	1	1179
4icb	1 (1998)	-5.5	10	5

Table S1. Structure Recognition: Discrimination of Native from Decoy Structures. Comparison of the performance of our potential (Native rank) with the performance of the potential derived by Feng (Feng, Kloczkowski, & Jernigan, 2007) and Krishnamoorthy (Krishnamoorthy & Tropsha, 2003).
